# Biceps Femoris Fascicle Lengths Increase after Hamstring Injury Rehabilitation to a Greater Extent in the Injured Leg

**DOI:** 10.1155/2022/5131914

**Published:** 2022-09-22

**Authors:** Rod Whiteley, Jack T. Hickey, Robin Vermeulen, Ryan Timmins, Thomas M. Best, Ebonie Rio, David Opar

**Affiliations:** ^1^Rehabilitation Department, Aspetar Sports Medicine Hospital, Doha, Qatar; ^2^School of Human Movement & Nutrition Sciences, The University of Queensland, Brisbane, Australia; ^3^School of Behavioural and Health Sciences, Australian Catholic University, Melbourne, Australia; ^4^Sports Performance, Recovery, Injury and New Technologies (SPRINT) Research Centre, Australian Catholic University, Fitzroy, Australia; ^5^Amsterdam, Academic Center for Evidence Based Medicine, Amsterdam IOC Center, ACHSS, Amsterdam, Netherlands; ^6^Department of Orthopedics, University of Miami Sports Medicine Institute, Miller School of Medicine, Coral Gables, FL, USA; ^7^La Trobe Sport and Exercise Medicine Research Centre, La Trobe University, Bundoora, Australia

## Abstract

**Objectives:**

Document changes in fascicle length during rehabilitation from hamstring injury of the injured and uninjured legs and secondarily to describe any association between these changes and reinjury rate.

**Design:**

Multicentre case series.

**Methods:**

Fifty-two prospectively included hamstring injured athletes had their biceps femoris long head fascicle lengths measured at the start and end of rehabilitation using two-dimensional ultrasound. Absolute and relative changes in fascicle length were compared for each leg using linear mixed models. Participants were followed for six months after being cleared to return to sport for any reinjury. Fascicle lengths and rehabilitation duration were compared for those who reinjured and those who did not.

**Results:**

Injured leg fascicle length was shorter at the start of rehabilitation (9.1 cm compared to 9.8 cm, *p* < 0.01 ) but underwent greater absolute and relative lengthening during rehabilitation to 11.1 cm (18% increase) compared to 10.2 cm (8% increase, *p* < 0.01 ) for the uninjured leg. There were no significant differences in any fascicle length parameter for the 5 participants who reinjured in the 6 months following their return to sport compared to those that did not reinjure.

**Conclusions:**

While both injured and uninjured legs displayed increases in fascicle length during rehabilitation, the larger fascicle length increases in the injured leg suggest that either a different training stimulus was applied during rehabilitation to each leg or there was a different response to training and/or recovery from injury in the injured leg. Reinjury risk appears to be independent of fascicle length changes in this cohort, but the small number of reinjuries makes any conclusions speculative.

## 1. Introduction

Hamstring strain injuries and reinjuries remain a leading cause of time-loss in sports that involve fast running [1, 2]. Biceps femoris long head fascicle length, as estimated using two-dimensional ultrasound imaging, appears to mediate the effects of both old age and history of hamstring injury on the risk of subsequent hamstring injury in a dose-response manner [3]. Shorter biceps femoris fascicle lengths are associated with higher rates of injury independent of these nonmodifiable risk factors [3]. Therefore, increasing biceps femoris fascicle length may be a key target of hamstring strain injury prevention and rehabilitation strategies. Before considering the effects of variations in rehabilitation approaches, it would be useful to understand what changes, if any, there are for the biceps femoris fascicle lengths of injured and uninjured legs during rehabilitation from hamstring strain injury.

Plausibly, the relative unloading associated with reductions in normal training and match exposure during recovery from injury may be associated with shortening of biceps femoris fascicle lengths as documented in the “washout” period of eccentric training studies [4]. Interestingly, several weeks of exposure to heavy load concentric exercise have been shown to shorten biceps femoris fascicles [4]. Typically heavy eccentric exercise has not been programmed during competition phases or during acute stages of rehabilitation [5–7], with previous expert recommendation specifically against this in early rehabilitation [8]. Recently, rehabilitation practices have shifted toward the earlier introduction of eccentric (lengthening) type exercises [9–11]. Increases in biceps femoris fascicle length have been observed in the injured leg of athletes during hamstring strain injury rehabilitation [9]. However, it is unknown if alterations in biceps femoris fascicle length also occur in the uninjured leg during rehabilitation, which may indicate if changes observed in the injured leg were a result of natural recovery after injury or adaptation to exercise. Regarding the assessment of muscle fascicle length, there are currently no commonly used methods which accurately assess the length of all fascicles in the biceps femoris long head [12]. However, the methodology [13] used in the current study has been prospectively associated with an increased risk of hamstring strain injury and is therefore of clinical interest.

Training studies in healthy adults show that relatively high-load eccentric exercise is a suitable stimulus to increase biceps femoris fascicle lengths [4, 14–16]. This stimulus likely exceeds the intensity of exercise that is performed during a typical rehabilitation program, at least for the injured leg. Eccentric overload appears to be a required stimulus for positive architectural adaptation of the hamstring muscles in healthy participants [17]. Training studies typically involve a minimum of 4 weeks, but more commonly 6 or more weeks' exposure to induce meaningful adaptations in architecture [18]. The median time to return to play from hamstring injury is reported to be in the order of approximately 2–4 weeks [19, 20], but injured athletes would likely only be capable of high-intensity eccentric overload exercise in the final stages of rehabilitation—perhaps a week or two at most, which suggests that there would be insufficient training time to induce architectural changes.

Research on the response of injured muscle to loading is sparse; however, a prodigious activation of satellite cells [21] and migration of nuclei [22] has been documented locally in response to muscle strain injury in vivo. The proliferation of satellite cells in the basement membrane of injured myofibres represents a very different environment to normal healthy muscle, which may respond differently to exercise loading. Plausibly the injured and uninjured legs may respond differently to loading that occurs during rehabilitation from hamstring strain injury. As these architectural differences are associated with injury risk, they may be associated with reinjury rates after returning to the sport. Accordingly, this exploratory study aimed to document the pre and postrehabilitation biceps femoris fascicle lengths in the injured and uninjured legs of a group of athletes across their rehabilitation period. Additionally, fascicle length and fascicle length changes during rehabilitation were examined for differences in those who suffered a reinjury within 6 months of returning to sport as a secondary aim.

## 2. Methods

Healthy adult male athletes across two different study sites who had a diagnosed hamstring strain injury were invited to take part in this study as part of two separate prospective studies (Ethics Approval IRB-A-AOSM-2020-003, and ACU Human Research Ethics Committee approval 2015-307H). Participants provided written informed consent prior to inclusion. Data from one of these studies have been published previously [9]. Participants did not overlap between the two studies. The initial diagnosis was made less than 7 days from the time of injury and was based on the following: the reported history of injury, including sudden onset of posterior thigh pain during activity, location of reported pain, positive pain on palpation, painful response to manual muscle testing, and was confirmed by diagnostic imaging if required/when available. Participants had their biceps femoris long head fascicle length assessed independently by one of two trained examiners (RGT and RV) using a previously documented method that has been demonstrated to have a minimum detectable change of 0.7 cm. The same examiner measured fascicle length again on the day of discharge from rehabilitation. Rehabilitation was supervised by a sports physiotherapist or clinical exercise physiologist in a criteria-based (not time-based) manner with an emphasis on the early introduction of eccentric exercise and progressive running as detailed in prior publications [9, 23] (Supplementary material). Athletes were required to report confidence in running and changing direction at full speed and during sports-specific activity simulations before being cleared to return to play. The athletes were monitored for a minimum of 6 months after their return to play for any reinjury.

Fascicle lengths of the biceps femoris long head were determined using two-dimensional ultrasound, which was extrapolated from pennation angle and muscle thickness [13]. This method has been validated against cadaveric hamstring muscles [24] and has demonstrated excellent interrater reliability [13]. Additionally, fascicle length using this measure has been shown to be associated, in a dose-response manner, with hamstring injury independent of age and history of injury [3]. While the measure is not truly reflective of all fascicles of the entire muscle (no currently available measures are), for convenience, in this paper, we will term this method as fascicle length. Two-dimensional images were saved and coded before analysis of the biceps femoris fascicle length was conducted offline by an examiner who was blinded to the injured leg at both measurement times and was blinded to the prerehabilitation measurement results at the time of the postrehabilitation final measurement.

Prerehabilitation fascicle lengths were compared using a linear mixed model regression with the participant as a random factor and leg (injured, uninjured) as a fixed factor. Where there were significant effects of prerehabilitation fascicle length, the relative change in fascicle length was compared using a mixed model with participants as random factors and legs (injured, uninjured) as fixed factors and an interaction effect of both applied. Postrehabilitation fascicle lengths were similarly compared using a mixed model with the participants as a random factor and fixed factors of the leg (injured, uninjured) and prerehabilitation fascicle length and the interaction of these two effects. Participants were followed for six months after discharge to document reinjury, which was defined as any hamstring injury in the same leg (irrespective of the muscle or the location). Exploratory logistic regression was conducted to examine any factors related to reinjury. The independent variables considered in these analyses were as follows: both the injured and uninjured leg's fascicle length at the start and end of rehabilitation, the relative and absolute change in the injured leg's fascicle length during rehabilitation, and the duration of rehabilitation (in days). Where the multiple logistic regression identified any significant factors, an analysis of variance was conducted to detect differences in these parameters for the reinjured participants. All models were checked by examining the distribution of the model's residuals, including frequency histograms with a fitted normal distribution, Q-Q plots, and Shapiro–Wilk estimation of the goodness of fit to a normal distribution. All statistical analyses were conducted in JMP pro v15.2.1 (SAS Institute Inc., Cary, NC, 1989–2021).

This research was funded, in part, by a grant from the Qatar National Research Foundation (NPRP92063036). The funding organisation took no part in the collection of data, their analysis and interpretation, nor did they have the right to approve or disapprove publication of the finished manuscript.

## 3. Results

This study prospectively included 52 athletes with a diagnosed hamstring strain injury. These athletes played Australian football (32), football (10), cricket (4), and futsal (3) and 1 athlete each played volleyball, hockey, and athletics. The median age was 25 (IQR: 20–29.75, range 18–39), and the mean height and weight were 181 cm (SD: 8.6 cm) and 83.1 kg (SD: 13.5 kg), respectively.

The athletes took a median of 16.5 days from their time of injury until the end of their rehabilitation (IQR: 12.25–20.75, range: 6–67) and the median time from the start to the end of rehabilitation was 13 days (IQR: 9–17, range 5–62).

**Figure 1:**
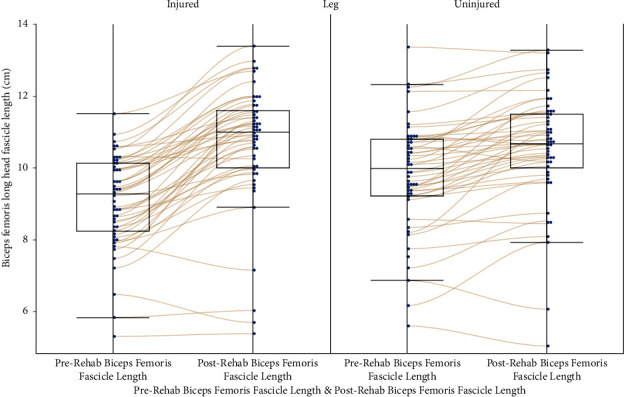
Pre and postrehabilitation biceps femoris fascicle lengths (cm) for the injured (left panel) and uninjured legs. Lines connect individual participants' measurements (blue dots).

**Figure 2:**
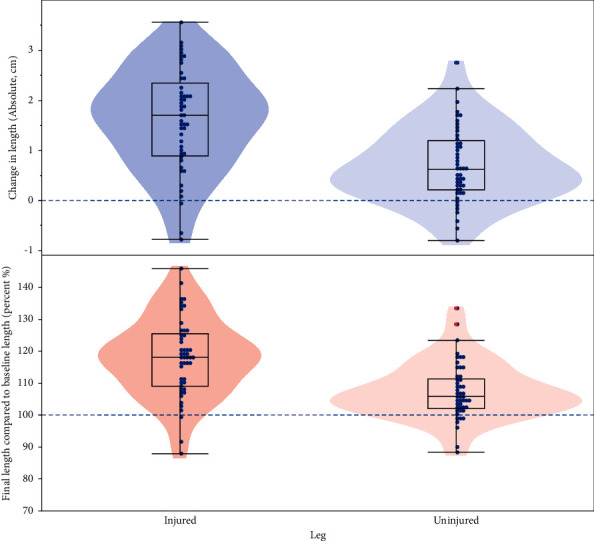
Absolute change of biceps femoris fascicle lengths in cm (upper panel) and relative changes for the injured (left side) and uninjured legs. Dashed horizontal lines represent no change for the absolute and relative comparisons (0 cm and 100%, respectively). Blue dots are individual observations, and overlying contour plots represent the distribution of observations.

Fascicle length data were missing from both the legs of 2 participants at prerehabilitation, both the legs for 1 participant at postrehabilitation and from the uninjured leg of 1 participant at postrehabilitation. At the start of rehabilitation, the uninjured legs had significantly longer biceps femoris fascicle lengths (9.8 cm (95% confidence interval: 9.4–10.2 cm)) compared to the injured legs (9.1 cm (8.7–9.5 cm), *p* < 0.0001, Figure 1). Both the injured and uninjured legs showed significant increases in their biceps femoris fascicle lengths during rehabilitation, with the injured leg showing a significantly greater improvement in relative length (18%, (15–21%) compared to 8% (5–11%), *p* < 0.0001, Figure 2) in the uninjured leg. At the end of rehabilitation, the injured legs showed significantly longer biceps femoris fascicle lengths (11.1 cm; 95% CI: 10.8–11.3 cm) compared to the uninjured legs (10.2 cm; 95% CI: 9.9–10.4 cm, *p* < 0.0001). The significant fixed effect parameter estimates were the leg (injured leg (0.45, (0.29–0.61), *p* < 0.0001) and prerehabilitation fascicle length (0.96, (0.82–1.10), *p* < 0.0001), but there was no significant interaction effect of these two factors (Table 1). All models were checked by analysing the residuals, specifically by plotting frequency histograms with a fitted normal distribution, normal quantile plots, and estimating fit with a Shapiro–Wilk test.

Five reinjuries were reported to have occurred in the same leg as the index injury within the 6 months' follow-up. Multiple logistic regression considering reinjury status identified the absolute change in the injured leg's fascicle length as significant (chi-square, *p*=0.0493). As the relative and absolute change in the fascicle length of the injured leg values are strongly correlated (adjusted *R*^2^ = 0.96, *p* < 0.0001), the regressions were repeated, removing the (nonsignificant) relative change. This resulted in the overall model no longer attaining the statistical significance, indicating the initial model was overfit.

## 4. Discussion

Biceps femoris fascicle lengths increased during rehabilitation from hamstring injury for almost every participant and legs, injured or uninjured, in this study. The prerehabilitation fascicle lengths were shorter in the injured leg, but the injured leg showed a significantly greater absolute and relative increase in length over the course of rehabilitation. This resulted in the injured legs showing slightly longer fascicles at the end of rehabilitation than the uninjured legs.

There may be 2 reasons for the differential response in the fascicle length of the injured and uninjured legs. The exercise intensity and volume were both likely different in each leg during rehabilitation, and it was plausible that the injured leg was exposed to a greater training stimulus. Additionally, it seems that injured muscle has the potential for an exaggerated response to exercise. Elevated gene levels of many components of the extracellular matrix are seen during remodelling [25–27], and the proliferation of myoblasts [21] and nuclear migration [22] within the myotube of regenerating muscle fibres suggests a fertile environment for muscle adaptation during loading. These changes have been documented 30 days after muscle injury—beyond the median time for return to play from hamstring strain injury—which suggests readaptation from injury is likely incomplete at this time in many injured athletes.

We were underpowered to detect any relationship between hamstring strain reinjury and fascicle length.

The minimum detectable change (MDC) for the 2D ultrasound method of estimating biceps femoris fascicle length is approximately 0.75 cm [13]. The average change in the fascicle length of the injured legs of participants was 1.6 cm (median 1.7 cm) and 0.71 cm (median 0.62 cm) for the uninjured legs. Forty-three of the participants had an increase in fascicle length in their injured leg that exceeded the MDC, whereas only 24 of the uninjured legs met this threshold. Plausibly, this may be a result of greater emphasis on exercise on the injured leg during rehabilitation. Alternatively, there may be a ceiling effect as the uninjured leg's fascicles were typically longer than the injured leg (40 of the 48 participants), yet at the end of rehabilitation, 28 out of 48 available participants had longer fascicles in their injured legs.

While nearly every participant showed an increase in fascicle length during rehabilitation, there were 3 uninjured and 6 injured legs in whom a reduction in fascicle length was observed, with 2 participants accounting for 4 of these measures. An analysis of the association between duration of rehabilitation and change in fascicle length was statistically significant; however, this is likely a spurious finding as one subject with, by far, the longest time to return to sport (67 days) was also the participant with the greatest reduction in fascicle length seen in the study. Exploratory secondary analysis excluding this participant showed no significant effect of rehabilitation duration on absolute or relative fascicle length change. Further research is required to confirm if there is a real effect of rehabilitation duration on fascicle length change. Plausibly, there is an association between rehabilitation duration and a participant's ability to perform resistance training of a sufficient intensity to induce fascicle lengthening [17]. Participants who are unable to perform heavy overload exercise during rehabilitation, perhaps due to pain or fear, may see their rehabilitation duration extended. If this were true, there should be an association between the volume of exercise performed and the duration of rehabilitation when matched for intensity. Future research could consider exercise dose; however, this will likely be a difficult undertaking as it will be difficult to quantify exercise between individuals in terms of absolute and relative intensity in the presence of injury.

There were no differences in any of the parameters of interest for those who did and did not suffer an reinjury (Supplementary Figures 1 and 2, Supplementary Table 1). The (small) magnitude of differences in all parameters of interest for those who did and did not suffer an reinjury that are likely below the MDC suggests that future research is unlikely to detect any clinically meaningful differences. The current study was likely underpowered to examine the effect of reinjury, with only 5 of the 52 participants suffering an reinjury during the follow-up period. This reinjury rate compares favourably with published data showing reinjury rates of up to 70% following hamstring strain injury rehabilitation [28]. The reinjury rates documented here are in the order of approximately 9-10%. For a primary study to have over 50 participants suffer a reinjury and therefore be adequately powered to detect the effect of a single independent variable over 700 hamstring injured athletes would need to be included [29]. A multivariate analysis would require more than 1,000 primary injured athletes to have enough power to detect significant factors associated with reinjury—more than an order of magnitude greater than what is typically seen in hamstring injury research. This underscores the importance of multicentre collaborative research in this area [30]. The current study used a follow-up period of 6 months for reinjury [7, 31]. Follow-up periods for hamstring reinjury research appear to range from 2 [32] to 12 [33] months. While the majority of reinjuries occur inside 6 months [33, 34], longer follow-up periods may have seen more reinjuries.

Although there were 7 missing fascicle length observations (as described in the results), the use of linear mixed models with subjects as random effects helps obviate this limitation. The measure of fascicle length is an estimation made from a validated equation [24]. This is due to the small transducer field of view being unable to capture an entire fascicle. However, whilst the results are still an estimation, the methodology and equation employed have been validated against cadaveric samples and show excellent agreement between dissection and estimation methods [24]. It is also the only current measure of muscle fascicle length that has been associated with injury risk [3]. Whilst recent work [35] has suggested that the current technique used for estimating fascicle length contains errors when compared to extended field of view (EFOV) ultrasound assessments, it should be noted that the EFOV assessment of biceps femoris long head fascicle length has not been validated against cadaveric data. Given the automated algorithms used to reconstruct EFOV scans and that the error associated with this reconstruction is typically unspecified, the lack of agreement between these two techniques is not indicative of the superiority of one approach. Given the existence of data comparing cadaveric tissue with the current technique of estimating fascicle length, we believe this to be a valid and robust approach. As is standard clinical practice, the exercise selection, frequency, duration, and intensity varied between participants. This likely influences the change in fascicle length seen during rehabilitation. To better understand the influence of exercise selection and dosage, future research might control for this prospectively during rehabilitation following hamstring injury.

## 5. Conclusion

Biceps femoris long head fascicle lengths increase during rehabilitation from hamstring strain injury by an amount that typically exceeds the minimum detectable change of the measurement. The increase in length seen is greater in the injured leg such that despite beginning rehabilitation with shorter fascicles, typically by the end of rehabilitation, the fascicle lengths are longer than that of the uninjured leg. There were no associations between any parameters and reinjury rates; however, all such analyses were underpowered due to the small number of reinjuries.

## 6. Practical Implications

- Most legs (injured and uninjured) showed increased fascicle length from the start to the end of rehabilitation. Repeated measures of fascicle length (instead of, for example, in the preseason only) are recommended to monitor for these changes, and practitioners should intervene accordingly (i.e., increase eccentric loading) when they are less than expected.
- The greater increase in fascicle length of the injured leg's hamstring suggests the healing environment may be more responsive to loading than the uninjured leg and underscores the importance of an active (exercise-based) approach to rehabilitation of hamstring injury
- The very small differences in all fascicle measures for those who reinjured compared to those who did not imply extremely large prospective studies will be required to detect any real effects in these parameters

## Figures and Tables

**Table 1:** Parameter estimates for the fixed effect linear mixed models.

Prerehabilitation biceps femoris fascicle length, parameter estimates, fixed effects							
Term	Estimate	Std. error	DF	*t* ratio	Prob>|t|	95% lower	95% upper
Intercept	9.43	0.19	49	50.27	<0.0001^*∗*^	9.06	9.80
Leg (injured)	−0.37	0.07	49	−5.25	<0.0001^*∗*^	−0.52	−0.23

Postrehabilitation biceps femoris fascicle length, fixed effects, parameter estimates							

Intercept	1.54	0.67	68.9	2.29	0.0253^*∗*^	0.20	2.90
Leg (injured)	0.45	0.08	51	5.65	<0.0001^*∗*^	0.29	0.61
Prerehab biceps femoris fascicle length	0.96	0.07	69.2	13.56	<0.0001^*∗*^	0.82	1.1
(Prerehab biceps femoris fascicle length-9.4)^*∗*^leg (injured)	0.08	0.06	54.1	1.42	0.1607	−0.03	0.19

Upper panel is the prerehabilitation biceps femoris fascicle length, denoting a significant effect of leg (injured leg shorter fascicles), and the lower panel shows the estimates for the postrehabilitation fixed effects: the injured leg with longer fascicles and a significant effect of baseline fascicle length, but no interaction effect. DF, degrees of freedom; Prob >|t|, probability that the observed statistic is greater than the absolute value of the *t* statistic; 95% lower, the lower limit of the 95% confidence interval of the estimate; 95% upper, the upper limit of the 95% confidence interval of the estimate.

## Data Availability

The data used to support this study are available from the corresponding author upon request.

## Supplementary Materials

Supplementary Materials Supplementary file 1: Rehabilitation protocols for the two sites. Supplementary file 2: Reinjury analysis—comparison of independent variables for those who did and did not reinjure after returning to sport.
