# Supplementary material for: Biceps Femoris Fascicle Lengths Increase after Hamstring Injury Rehabilitation to a Greater Extent in the Injured Leg
Source: Transl Sports Med. 2022 Sep 22;2022:5131914. doi: 10.1155/2022/5131914 (PMC11022767; doi:10.1155/2022/5131914)
Supplement: Supplementary Materials — Supplementary file 1: Rehabilitation protocols for the two sites. Supplementary file 2: Reinjury analysis—comparison of independent variables for those who did and did not reinjure after returning to sport. [file 5131914.f1.zip › Supplementary Figures and Table.docx]

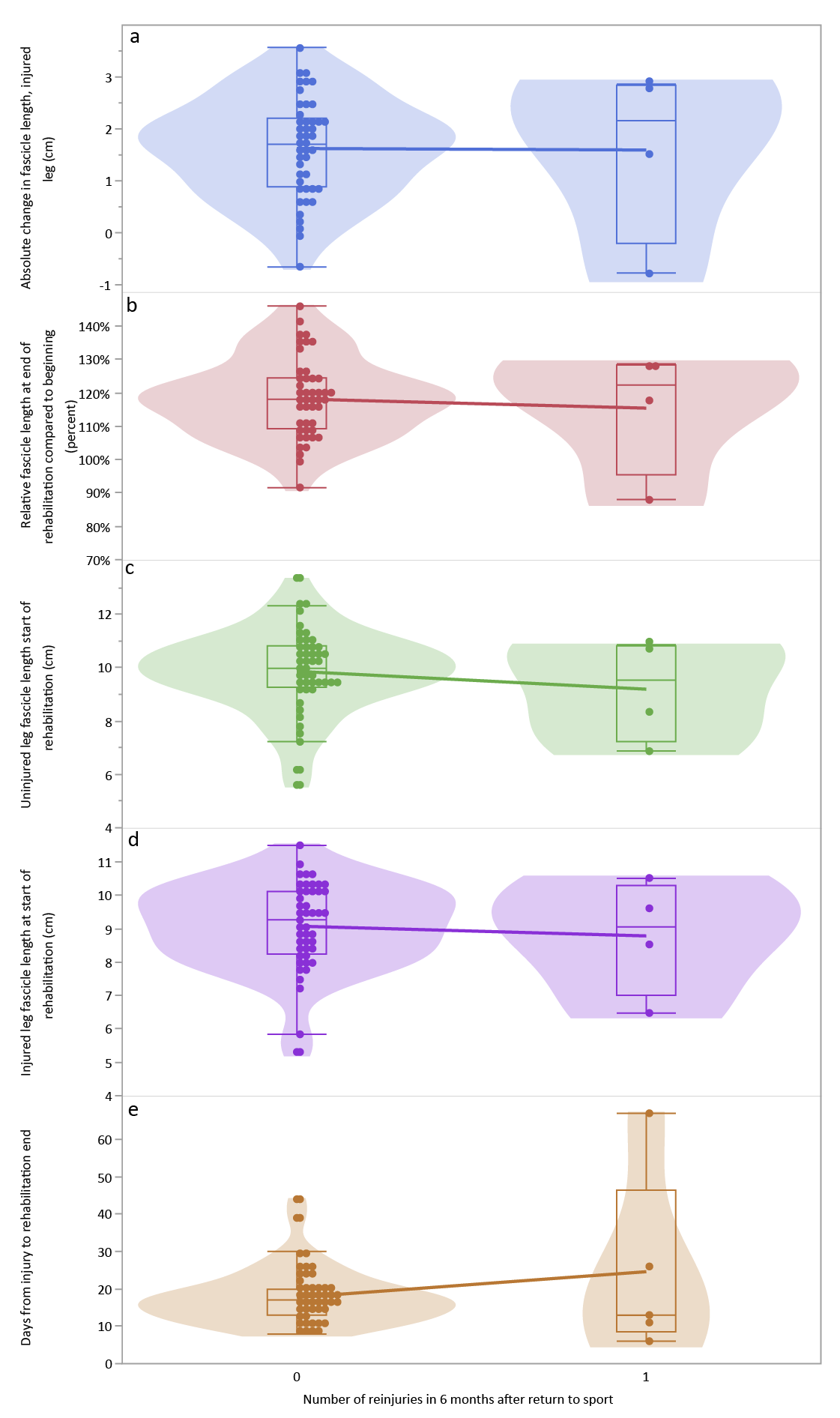


Supplementary Figure 1: Analysis of reinjury – left side is no reinjury (0) right side 1 reinjury in the 6 months following return to sport. Top 2 panels (a and b) are the absolute (cm) and relative (percent) fascicle length changes respectively, third and fourth panels (c and d) are the fascicle length at the start of rehabilitation for the uninjured and injured legs respectively (cm), and the bottom panel (e) is the total days in rehabilitation. No significant differences were found for any of the parameters of interest in those who suffered a reinjury compared to those who didn’t (Welch’s unequal variance t-test all p>0.05). Parameter estimates and between group comparisons for fascicle lengths and rehabilitation duration are shown in Supplementary Table 1


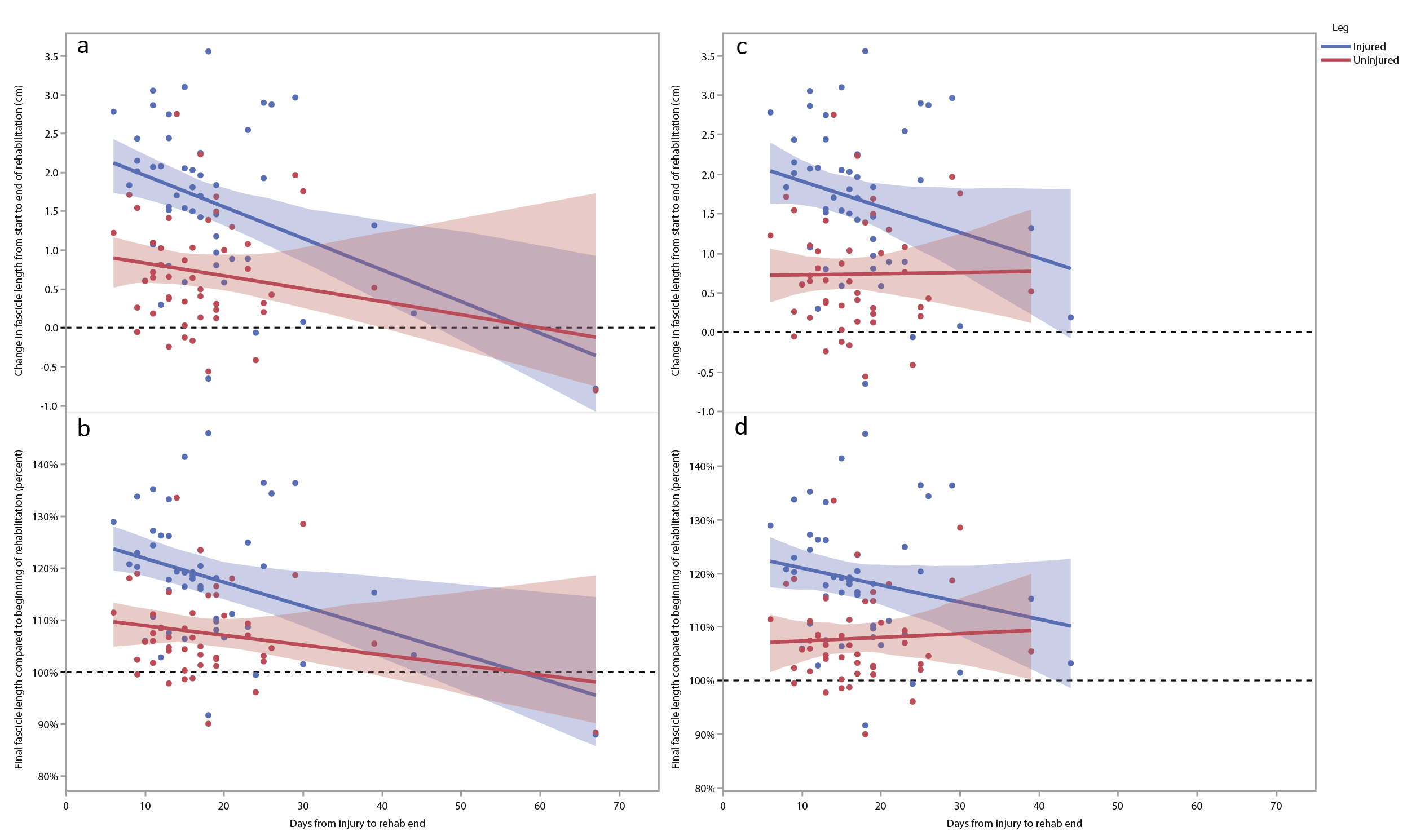


Supplementary Figure 2: Rehabilitation duration compared with absolute change in fascicle length (upper panel, graphs a and c) and relative change in fascicle length (lower panel, b and d) for the injured (red) and uninjured (blue) legs with lines of best fit and 95% confidence intervals. Left panels are for all participants, with line of best fit suggesting a weak negative trend of increasing days in rehabilitation to be associated with shorter fascicles however right panel is the same analysis with one outlying participant (return to sport duration = 67 days) removed suggesting this participant’s data are exerting leverage on the regression.

Supplementary Table 1: Reinjury analysis. Fascicle length at the start and end of rehabilitation for the injured and uninjured leg, the absolute and relative change in fascicle length (relative change expressed as final length divided by initial length), and days from injury to return to play for those who did and didn’t suffer a reinjury. Std Dev: Standard deviation. Std Err Mean: Standard Error of the Mean *p-value is the result of Welch's unequal variances t-test.

|  | Number | **Mean** | *Std Dev* | Std Err Mean | 95% Confidence Interval | p-value* |
| --- | --- | --- | --- | --- | --- | --- |
| Injured leg fascicle length pre rehabilitation | | | | | | |
| No reinjury | 46 | **9.08** | *1.27* | 0.19 | 8.71 to 9.46 | 0.76 |
| Reinjury | 4 | **8.79** | *1.74* | 0.87 | 6.02 to 11.56 |  |
| Uninjured leg fascicle length pre rehabilitation | | | | | | |
| No reinjury | 46 | **9.86** | *1.51* | 0.22 | 9.41 to 10.31 | 0.54 |
| Reinjury | 4 | **9.19** | *1.93* | 0.96 | 6.12 to 12.26 |  |
| Injured leg absolute change (cm) | | | | | | |
| No reinjury | 45 | **1.62** | *0.94* | 0.14 | 1.34 to 1.91 | 0.98 |
| Reinjury | 4 | **1.60** | *1.70* | 0.85 | -1.11 to 4.30 |  |
| Uninjured leg absolute change (cm) | | | | | | |
| No reinjury | 44 | **0.74** | *0.73* | 0.11 | 0.51 to 0.96 | 0.46 |
| Reinjury | 4 | **0.37** | *0.85* | 0.43 | -0.99 to 1.72 |  |
| Injured leg relative length change (final length/initial length) | | | | | | |
| No reinjury | 45 | **1.18** | *0.12* | 0.02 | 1.15 to 1.22 | 0.79 |
| Reinjury | 4 | **1.15** | *0.19* | 0.09 | 0.85 to 1.46 |  |
| Uninjured leg relative final length change (final length/initial length) | | | | | | |
| No reinjury | 44 | **1.08** | *0.09* | 0.01 | 1.05 to 1.11 | 0.37 |
| Reinjury | 4 | **1.03** | *0.10* | 0.05 | 0.87 to 1.18 |  |
| Days from injury to return to play | | | | | | |
| No reinjury | 47 | **17.72** | *7.35* | 1.07 | 15.56 to 19.88 | 0.57 |
| Reinjury | 5 | **24.60** | *24.83* | 11.10 | -6.22 to 55.42 |  |
