# Supplementary material for: Biceps Femoris Fascicle Lengths Increase after Hamstring Injury Rehabilitation to a Greater Extent in the Injured Leg
Source: Transl Sports Med. 2022 Sep 22;2022:5131914. doi: 10.1155/2022/5131914 (PMC11022767; doi:10.1155/2022/5131914)

Rehabilitation protocol Site A

| **Stage** | **Content** | **Criteria to progress** |
| --- | --- | --- |
| Stage 1 | *All activity to be pain-free*  **2 leg squat, or if able, Single leg squat**  Maintain pelvis control, hip and knee alignment, Squat to 45°, hold, return to start.  **Supine Bridge – 2 leg**  2 sec up, 2 sec down (4 sec total per rep.) Begin at 45°. Must reach knee-hip-shoulder in alignment. 4 X 15  **Supine Isometric Heel digs**  In supine, **painlessly** pull heel into bed through range. Can bias with tibial IR/ER when painless.  **Exercise Bike**  Upright or recumbent, can substitute with elliptical trainer.  **Isometric manual resisted hamstring**  Therapist applied resistance **isometrically** in varying angles in prone.  **Soft tissue massage**  Proximal and distal to injury site, lymphatic drainage.  **Active range of motion exercises**  Supine active knee flexion & extension then Prone active flexion & extension. | Criteria to progress to Stage 2:  1. Painless Single Leg Squat. 2. Painless Bike, 150W 5 minutes. 3. Full Knee Extension Supine. |
| Stage 2 | *Any exercise from Stage 1 permitted, additionally:*  **Supine Bridge - 1 leg**  Same rate as for 2 legs, other knee in full Extension, thighs parallel throughout exercise. 4 X 15  **Walk-Jog**  Walk 20m corners, jog the 30m straight, painless. Begin at 25% (self-rated) jog, progress to max 70%.  **Triple Extension Walk**  100m laps, every third step triple extension – i.e. alternating legs.  **“A” drill**  Walking late swing knee extension, painless. Alternating legs, 100m lap. Soft tissue massage Can massage injured area. Maximum allowed pain VAS: 4/10. Therapist uses caution with any report of discomfort, monitor symptoms, adjust accordingly. Stretching Hamstring (supine, 90° hip Flexion, knee Extension);  SLR (Supine to onset of discomfort add ankle DF)  Initially active, patient-controlled, progress to passive, end range. SLR mobilisation if indicated. Resisted hamstring Note tibial rotation as indicated. 4 X 15 repetitions, aiming for fatigue | *Criteria to progress to Stage 3:*   1. Run ≥ 70% Patient-rated. 2. ROM Hamstrings ≥ 75% uninvolved side. 3. ROM SLR ≥ 75% uninvolved side. |
| Stage 3 | *Any exercises from stages 1 and 2, additionally:*  **Single Leg Bridge**  1 sec repetition, 2 sec recovery. 4 X 8 repetitions.  **Single Leg Bridge, foot on Swiss Ball**  2 sec up, 2 sec down. 4 X 8 repetitions.  **Interval running**  20m jog 30m run. Begin running at 70% (patient rated), progressing by 10% steps, painlessly. At 90%, progress by 5%. Monitor performance by hand timing.  **Modified T-Drill**  Direction changing running over T-Drill course. Begin at patient-rated 70%, progress as able by 10% until 90%, then by 5%. Monitor performance by hand timing.  **Eccentric Exercises**  Nordic Hamstrings, manual resisted eccentric, prone catches, Arabesque (single leg stance, trunk flexion), | Criteria to progress to Stage 4 (Sport Specific Rehab):  1. 100% running speed. 2. Painless high speed direction changes. |
| Stage 4 | *Any exercises from stages 1-3, additionally on-field, football specific drills:* Direction Change drills With and without the ball, 40 mins Jumping drills 10-15 minutes | *Criteria to progress to Stage 5 (Sport Specific Rehab):*  1. Painless completion of stage 4. |
| Stage 5 | **Passes and run**  **Long passes progression**  **Crosses (static)**  **Corner Kicks**  **Crosses (dynamic)** | *Criteria to progress to Stage 6 (Sport Specific Rehab):*  1. Painless completion of stage 5. |
| Stage 6 | **Passes and run**  **Shooting scenarios**  **Competitive 1 versus 1 drills**  **Shooting scenarios**  **Scoring scenarios** | *Criteria to progress to medical review for return to sport:*  1. Painless completion of stage 6. |

Rehabilitation protocol site B

Intensity and distance of the nine-stage progressive running protocol. Walk is defined as regular gait, jog as less than 50% of perceived maximal running speed, run as less than 70% of perceived maximal running speed and sprint as greater than 90% of perceived maximal running speed. All participants commenced at stage 1 when they could walk with normal gait within pain-limits.

| **Stage** | **Acceleration** | **Hold** | **Deceleration** |
| --- | --- | --- | --- |
| 1 | Walk 20m | Jog 10m | Walk 20m |
| 2 | Walk 15m | Jog 20m | Walk 15m |
| 3 | Walk 10m | Jog 30m | Walk 10m |
| 4 | Jog 20m | Run 10m | Jog 20m |
| 5 | Jog 15m | Run 20m | Jog 15m |
| 6 | Jog 10m | Run 30m | Jog 10m |
| 7 | Run 20m | Sprint 10m | Run 20m |
| 8 | Run 15m | Sprint 20m | Run 15m |
| 9 | Run 10m | Sprint 30m | Run 10m |


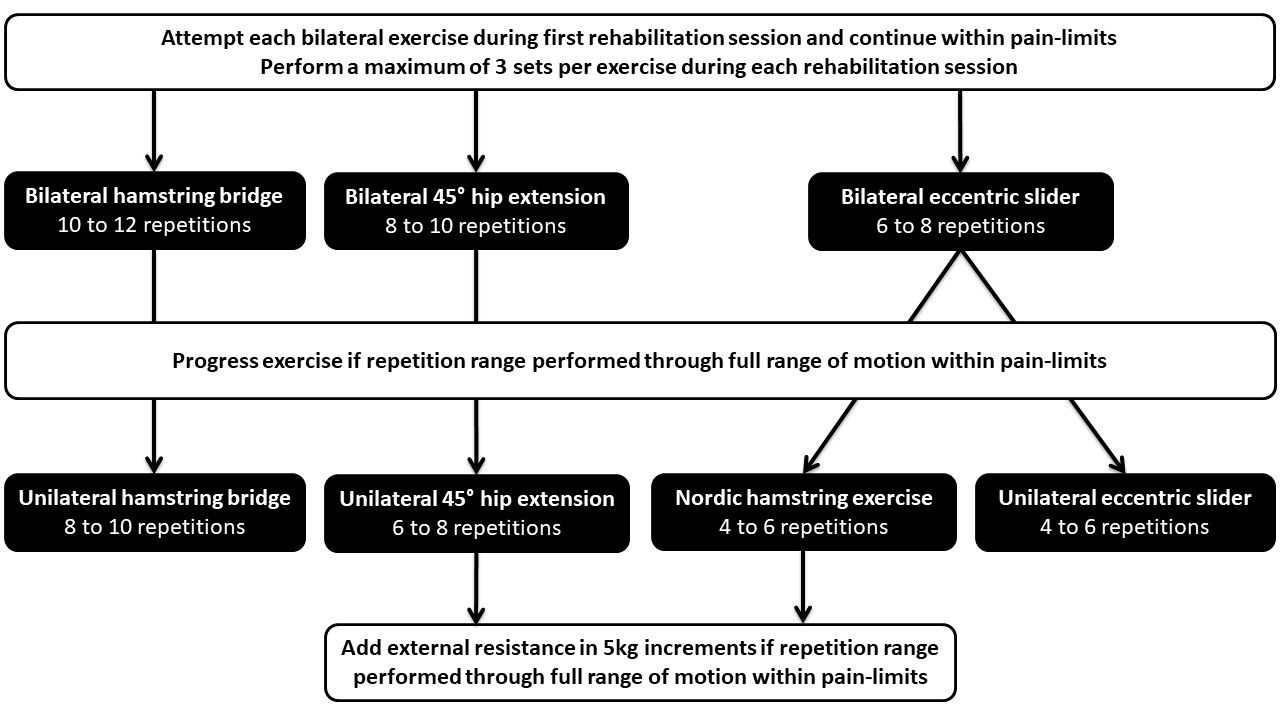

Supplement: Supplementary Materials — Supplementary file 1: Rehabilitation protocols for the two sites. Supplementary file 2: Reinjury analysis—comparison of independent variables for those who did and did not reinjure after returning to sport. [file 5131914.f1.zip › Supplementary File - Rehabilitation protocols.docx]
